# Awareness and knowledge about human papillomavirus vaccination and its acceptance in China: a meta-analysis of 58 observational studies

**DOI:** 10.1186/s12889-016-2873-8

**Published:** 2016-03-03

**Authors:** Yanru Zhang, Ying Wang, Li Liu, Yunzhou Fan, Zhihua Liu, Yueyun Wang, Shaofa Nie

**Affiliations:** Department of Epidemiology and Biostatistics, School of Public Health, Tongji Medical College, Huazhong University of Science and Technology, Wuhan, China; Shenzhen Maternity and Child Health Hospitals, Shenzhen, Guangdong P. R. China; Mental Health Center, Renmin Hospital of Wuhan University, Hubei Provincial Mental Health Center Wuchang District, Wuhan, Hubei P. R. China

**Keywords:** Awareness, Acceptance, Human papillomavirus vaccines, Cervical cancer, Meta-analysis

## Abstract

**Background:**

The human papillomavirus (HPV) vaccines have been widely introduced in immunization programs worldwide, however, it is not accepted in mainland China. We aimed to investigate the awareness and knowledge about HPV vaccines and explore the acceptability of vaccination among the Chinese population.

**Methods:**

A meta-analysis was conducted across two English (PubMed, EMBASE) and three Chinese (China National Knowledge Infrastructure, Wan Fang Database and VIP Database for Chinese Technical Periodicals) electronic databases in order to identify HPV vaccination studies conducted in mainland China. We conducted and reported the analysis in accordance with the Preferred Reporting Items for Systematic Reviews and Meta-Analyses (PRISMA) guidelines.

**Results:**

Fifty-eight unique studies representing 19 provinces and municipalities in mainland China were assessed. The pooled awareness and knowledge rates about HPV vaccination were 15.95 % (95 % CI: 12.87–19.29, *I*^*2*^ = 98.9 %) and 17.55 % (95 % CI: 12.38–24.88, *I*^*2*^ = 99.8 %), respectively. The female population (17.39 %; 95 % CI: 13.06–22.20, *I*^2^ = 98.8 %) and mixed population (18.55 %; 95 % CI: 14.14–23.42, *I*^2^ = 98.8 %) exhibited higher HPV vaccine awareness than the male population (1.82 %; 95 % CI: 0.50–11.20, *I*^2^ = 98.5 %). Populations of mixed ethnicity had lower HPV vaccine awareness (9.61 %; 95 % CI: 5.95–14.03, *I*^2^ = 99.0 %) than the Han population (20.17 %; 95 % CI: 16.42–24.20, *I*^2^ = 98.3 %). Among different regions, the HPV vaccine awareness was higher in EDA (17.57 %; 95 % CI: 13.36–22.21, *I*^2^ = 98.0 %) and CLDA (17.78 %; 95 % CI: 12.18–24.19, *I*^2^ = 97.6 %) than in WUDA (1.80 %; 95 % CI: 0.02–6.33, *I*^2^ = 98.9 %). Furthermore, 67.25 % (95 % CI: 58.75–75.21, *I*^*2*^ = 99.8 %) of participants were willing to be vaccinated, while this number was lower for their daughters (60.32 %; 95 % CI: 51.25–69.04, *I*^*2*^ = 99.2 %). The general adult population (64.72 %; 95 % CI: 55.57–73.36, *I*^2^ = 99.2 %) was more willing to vaccinate their daughters than the parent population (33.78 %; 95 % CI: 26.26–41.74, *I*^2^ = 88.3 %). Safety (50.46 %; 95 % CI: 40.00–60.89, *I*^*2*^ = 96.6 %) was the main concern about vaccination among the adult population whereas the safety and efficacy (68.19 %; 95 % CI: 53.13–81.52, *I*^*2*^ = 98.6 %) were the main concerns for unwillingness to vaccinate their daughters.

**Conclusions:**

Low HPV vaccine awareness and knowledge was observed among the Chinese population. HPV vaccine awareness differed across sexes, ethnicities, and regions. Given the limited quality and number of studies included, further research with improved study designis necessary.

**Electronic supplementary material:**

The online version of this article (doi:10.1186/s12889-016-2873-8) contains supplementary material, which is available to authorized users.

## Background

Cervical cancer, one of the most common cancers observed in females [1], affects more than 529,000 annually around the world [2]. More than 85 % of the global cervical cancer burden occurs in developing countries [2], with 75,500 incidences reported annually in China. Human Papillomavirus (HPV) infection is the most important risk factor for cervical cancer [3]. Although a single HPV infection can easily be eliminated through the immune system, malignant transformation of cervical epithelial cells may be induced in a small proportion of women affected by persistent virus infection.

Vaccines have always been among the most effective interventions for infectious diseases [4]. Prophylactic vaccines of cervical cancer manufactured by Merck &Co. have been approved by FDA and have been commercially available since 2006 [5]. The approval of vaccines for the HPV increased the possibility of eradicating cervical cancer in the near future. However, it is noteworthy that awareness of HPV and the general attitude towards vaccination were crucial factors for acceptance of vaccination among the population. In addition, increasing number of studies addressing the hesitation to get vaccinated have been conducted in the recent years, portraying the challenging and dynamic period of indecisiveness concerning HPV vaccination [6].

The HPV vaccine has been widely introduced in the vaccination programs of Hong Kong, however, is not popularly accepted in Mainland China at present. In addition, despite the numerous published studies focusing on the topic of HPV and vaccination in recent years, there is no comprehensive information concerning the acceptance and obstacles associated with vaccination among the population of Mainland China. In order to develop a practical vaccination program in the future, it is imperative to assess the level of awareness and knowledge about HPV, and the general attitude towards HPV vaccination among the Chinese population, as they are important behavioral determinants that will ultimately affect the acceptance of vaccination among the Chinese population. Therefore, we conducted a meta-analysis in order to gain a better understanding of this issue that may help generate new ideas to make future generalization of HPV vaccination possible in China.

## Methods

### Search strategy

The meta-analysis was conducted in compliance with the Preferred Reporting Items for Systematic Reviews and Meta-Analyses (PRISMA) guidelines [7]. The Chinese literature was searched using the China National Knowledge Infrastructure (CNKI), Wan Fang Database and VIP Database for Chinese Technical Periodicals (VIP) using the keywords “HPV vaccine OR cervical vaccine”. The literature in English was searched using PubMed and EMBASE, and relevant studies were identified with the search terms “HPV OR cervical cancer” AND “vaccine OR vaccination OR immunization” AND “awareness OR knowledge OR acceptability OR acceptance OR willingness OR perception OR attitude OR recognition” AND “China OR Chinese.” The publication time was limited to 2006–2015, as HPV vaccine was introduced in the world in 2006. Data retrieval was supplemented by manually searching for the reference list of key reviews and references from retrieved studies. No language restriction was imposed.

### Selection criteria

The inclusion criteria for the epidemiological studies were the following: (1)study involved at least one of the key terms “HPV vaccine awareness”, “knowledge”, and “acceptability”for any region of Mainland China (excluding studies conducted in Taiwan, Hong Kong and Macao due to differencesin socio-economic levels and health policies between these regions and Mainland China), (2) original data was available regardless of whether it was obtained directly from the article or traced from secondary data in the article. Studies that examining the effects of health educational interventions were excluded.

### Data extraction

A data abstraction form was constructed after scanning the selected articles. For each included study, we extracted the following information: author, publication year, region, study instrument, study subject (age, sex and ethnicity), sampling method, sample size (N), the number of participants for the assessment of HPV vaccine awareness, knowledge, and acceptance, or the rate percentage proportions for these studied factors. We also extracted the reasons for unwillingness to be vaccinated if this information was available. The number of studied cases(n) and sample size(N) were the two necessary parameters for the calculation of the pooled rates of HPV vaccine awareness, knowledge, and acceptance of vaccination in the meta-analysis. In particular, the number of studied cases (n) was obtained directly from the original studies or by multiplying the sample sizes (N) with the proportions (%) associated with the investigated factors reported in the original studies.

### Quality assessment

We employed a flexible appraisal scale suggested by Iain Crombie [8] for the assessment of the quality of cross-sectional studies. The scale contains seven indexes: (1) design is scientific, (2) data collection strategy is reasonable, (3) sample response rate is reported, (4) samples can represent the general population well, (5) the research purpose and method is reasonable, (6) the test efficiency is reported, (7) the statistical method is reasonable. For each index, the study was scored “1,” “0,”or “0.5” for “yes,” “no,”or “unclear,” respectively. The maximum score in the scale is 7 points, with scores of 6.0–7.0 points as grade A, scores of 4.0–5.5 points as grade B, and scores of less than 4.0 points as grade C.

### Data analysis

We used “rate” to evaluate the studied items. The rate for HPV vaccine awareness was calculated by dividing the number of cases who were aware of HPV vaccine (n1) by the sample size (N); the rate for HPV vaccine knowledge was calculated by dividing the number of cases who knew the relationship between HPV (vaccine) and cervical cancer (n2) by the sample size (N); the rate for acceptance to be vaccinated was calculated by dividing the number of cases who were willing to get vaccinated (n3) by the sample size (N); the rate for acceptance of parents to vaccinate their daughters was calculated by dividing the number of cases who were willing to vaccinate their daughters (n4) by the sample size(N); the rate for reasons of unwillingness to be vaccinated was calculated by dividing the number of cases who gave a reason (n5) by the number of cases who were unwilling to be vaccinated (N-n3).

Meta-analysis was conducted using a random effects model. Given the requirement for normalization of single rate in meta-analysis, an arcsine transformation for the original rate was performed to meet the requirement [9]. Statistical heterogeneity among the studies was estimated by Chi-square test at the significance level of *P* < 0.10, and using the I-square (*I*^*2*^) statistic to quantify the heterogeneity of the results. Publication bias was detected by Egger’s test (*P* < 0.05 was considered statistically significant) [10]. R statistical software (Version 2.11.1) was used for all the calculations.

### Consent statement

As this study was a meta-analysis, we did not include any humans and animals. This study was approved by the Ethics Committee of Huazhong University of Science and Technology.

## Results

### Screening process

**Fig. 1:**
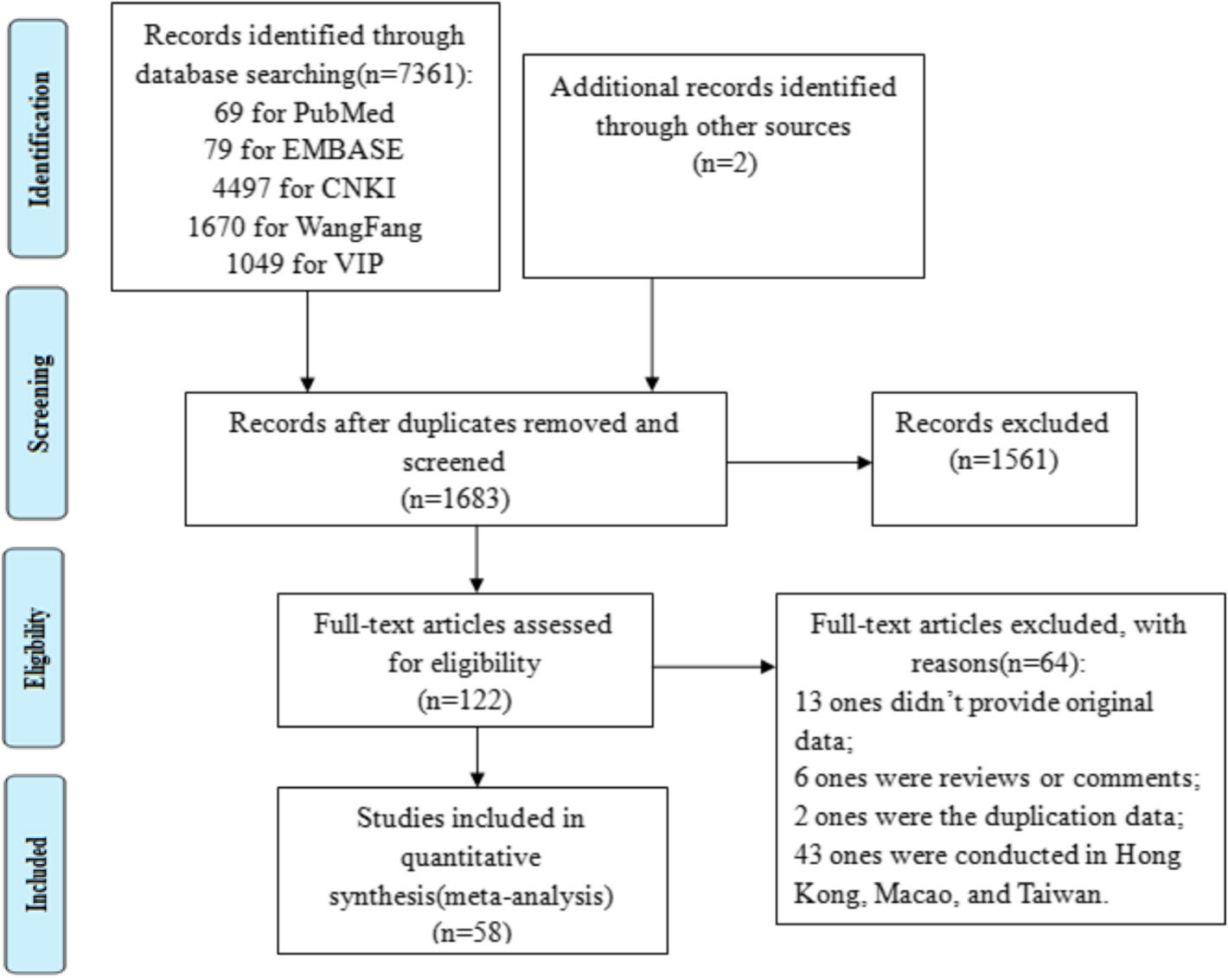
PRISMA Flow Diagram for Identification of Studies for Meta-analysis

Our search returned 1683 articles. A flow diagram of the selection process is shown in Fig. 1. Of the original articles, 1561 articles that were not clearly relevant to the analysis were excluded. After diligently reading the full text of the remaining 122 studies, 64 studies were excluded because they did not meet the inclusion criteria. Consequently, 58 observational studies [11–68] were included for the meta-analysis.

### Study characteristics

We included 58 individual studies [11–68] representing 19 provinces and municipalities in Mainland China (Table 1). Eighty-three thousand, seven hundred and five participants were interviewed, the majority of which were females. Nearly all the studies were published after 2009, and 38 studies were published in the recent three years. A questionnaire survey was conducted for all the studies included in the analysis, 12 of which were interview-administered, while nine were self-administered questionnaires (Table 1). After conducting a quality assessment on the included studies, 51 studies were categorized as grade A, and seven as grade B (Table 2).

**Table 1:** Characteristics of included studies

Study	Region^a^	Study instrument^b^	*N*	Population (age)			F/(F + M)	Sampling method	Ethnicity
				Range	$\overline{\mathrm{x}}\pm \mathrm{s}$	Group^c^			
Huang He, 2013 [33]	CLDA	Q	470	NA	20.09 ± 1.33	CS	0.504	Randomized	Han
Ma Xiaojing, 2013 [12]	EDA	IAQ	1451	NA	45.1 ± 10.8	A	1	Convenience	Mixed
He Mei, 2011 [41]	CLDA	Q	10,611	18–82	38.02 ± 9.57	A	1	Convenience	Han
Cui Bo, 2010 [13]	EDA	IAQ	1160	15–59	35.66 ± 11.72	A	1	Randomized	Mixed
He Xin, 2010 [29]	NA	SAQ	903	16–26	19.14 ± 1.01	CS	0.52	Cluster	Han
Xu Jing, 2014 [11]	CLDA	Q	353	18–24	20.96	CS	0.683	Cluster	Han
Feng Suwen, 2010 [46]	EDA	Q	1432	18–50	35.3	A	1	Cluster	Han
Yan Jun, 2013 [43]	WUDA	IAQ	1681	30–49	NA	A	1	Cluster	Han
Long Xiange, 2011 [21]	EDA	Q	286	NA	18.5	CS	NA	Convenience	Han
Hu Haishan, 2014 [28]	EDA	Q	542	31–60	41.57 ± 5.77	P	0.685	Cluster	Mixed
Wu Ying, 2011 [25]	EDA	IAQ	489	15–50	NA	A	1	Randomized	Han
Li Juan, 2011 [14]	EDA	IAQ	160	NA	36.55 ± 9.59	A	0.738	Randomized	Mixed
Fan Baojian, 2009 [23]	EDA	Q	962	19–72	43.38 ± 8.29	A	1	Cluster	Mixed
Xiao Wei, 2009 [45]	NA	Q	378	21–74	36.19	A	1	Convenience	Han
Wang Xuemin, 2012 [15]	WUDA	IAQ	2269	25–73	43.54 ± 7.67	A	1	Cluster	Han
Shao Shujuan, 2013 [17]	EDA	Q	594	≤60	36.02 ± 10.54	A	1	Randomized	Han
Xu Wenyu, 2013 [18]	EDA	Q	3000	20–30		A	1	Convenience	Han
Ma Dong, 2013 [40]	NA	Q	258	17–24	19.23 ± 0.89	CS	0.55	Cluster	Han
Zhou Lixia, 2011 [42]	EDA	IAQ	752	16–55	NA	A	1	Randomized	Han
Huang Yanhua, 2014 [39]	EDA	Q	378	15–50	NA	A	0.5	Randomized	Han
Wang Haiqiu, 2011 [31]	CLDA	Q	257	20–53	33.6 ± 0.5	A	1	Randomized	Han
Ma Dong, 2012 [30]	CLDA	Q	198	20–54	31.8 ± 7.0	A	0.89	Convenience	Han
Yao Chenglian, 2012 [19]	EDA	Q	1198	16–65	NA	A	1	Convenience	Han
Xamxinuer Ablimit, 2009 [26]	WUDA	Q	245	23–85	48.8	A	1	Convenience	Mixed
Xu Lina, 2013 [22]	NA	Q	1666	15–59	NA	A	1	Randomized	Han
Zhang Hui, 2014 [37]	CLDA	Q	341	32–50	39.56 ± 3.47	P	0.63	Cluster	Han
Yu Jing, 2013 [44]	CLDA	Q	750	15–59	35.75 ± 9.4	A	1	Randomized	Han
Guzalnur Abduxkur, 2012 [32]	WUDA	IAQ	560	NA	NA	A	0	Convenience	Mixed
Cai Jing, 2013 [24]	WUDA	Q	648	NA	NA	A	0	Randomized	Mixed
Li Li, 2010 [27]	WUDA	Q	1989	16–59	NA	A	1	Cluster	Mixed
Ying Wen, 2014 [35]	CLDA	Q	1878	17–25	NA	CS	0.679	Randomized	Mixed
Zhang Shaokai, 2013 [38]	NA	Q	2895	NA	40.4 ± 4.68	P	0.628	Cluster	Mixed
Wang Shaoming, 2014 [20]	NA	Q	3368	NA	19.82 ± 1.31	CS	0.51	Randomized	Mixed
Yan Hong, 2013 [16]	EDA	SAQ	360	18–36	25.1 ± 3.5	A	1	Convenience	Mixed
Li Jing, 2009 [34]	NA	Q	6024	14–59	34.6 ± 1.7	A	1	Cluster	Han
Zhao Fanghui, 2012 [36]	NA	Q	11,681	NA	34 ± 11.8	A	0.705	Randomized	Han
Ayizuoremu · mutailipu, 2015 [64]	WUDA	Q	1900	16–60	NA	A	1	Cluster	Mixed
Zeng Xiaomin, 2015 [55]	EDA	SAQ	2004	NA	NA	C	1	Cluster	Han
Wang Ling, 2015 [49]	CLDA	Q	125	18–23	20.5	C	1	Convenience	Han
Liu Qiong, 2015 [57]	CLDA	Q	590	14–20	15.34 ± 1.3	C	0.91	Convenience	Han
She Qian, 2015 [59]	EDA	IAQ	209	19–45	NA	A	1	Randomized	Han
Chen Ling, 2015 [50]	NA	IAQ	300	21–28	24 ± 0.8	C	1	Randomized	Han
Cheng Lihong, 2015 [51]	EDA	Q	1256	19–55	NA	A	1	Convenience	Han
Zhu Qiaoyang, 2015 [58]	EDA	Q	362	18–66	42.2 ± 6.3	A	1	Convenience	Han
Lei Juhong, 2015 [61]	EDA	Q	300	15–64	NA	A	NA	Randomized	Han
Zhao Bixia, 2015 [63]	CLDA	Q	138	25–50	NA	A	NA	Convenience	Han
Xie Wenliu, 2015 [48]	CLDA	Q	192	15–70	NA	A	0.51	Convenience	Han
Zhou Yanqiu, 2015 [47]	EDA	SAQ	600	≥21	NA	A	NA	Convenience	Han
Meng Liping, 2015 [60]	EDA	SAQ	1652	20–65	38.09 ± 8.21	A	NA	Cluster	Han
Zou Huachun, 2015 [53]	EDA	SAQ	368	NA	NA	A	0	Convenience	Han
Zou Huachun, 2015 [52]	EDA	SAQ	351	16–25	NA	C	0.524	Cluster	Mixed
Gu Can, 2015 [62]	CLDA	Q	117	19–23	20.8 ± 1	C	1	Convenience	Han
Wang Wei, 2015 [56]	EDA	Q	360	NA	41.77 ± 3.33	P	0.522	Cluster	Mixed
Abida Abudukadeer, 2015 [65]	WUDA	IAQ	5000	20–51	NA	A	1	Convenience	Mixed
Zhang Shaokai, 2015 [54]	CLDA	Q	2895	NA	40.4 ± 4.68	P	0.628	Cluster	Mixed
Pan Xiongfei, 2015 [66]	CLDA	Q	1878	17–25	20.8 ± 1.3	C	0.679	Convenience	Mixed
Fu Chunjing, 2015 [67]	CLDA	SAQ	605	18–26	21.6 ± 1	C	0.689	Randomized	Mixed
Hu Shangying, 2105 [68]	EDA	IAQ	316	18–25	23.2 ± 1.7	C	1	Cluster	Han

^a^ *EDA* eastern developed areas, such as Beijing (city), Tianjin (city), Shanghai (city), Dalian (city), Shandong(province), Jinan(city), Zhejiang(province), Hangzhou(city), Ningbo(city), Jiangsu(province), Wuxi(city), Guangdong(province), Guangzhou(city), Shenzhen(city), Dongguan(city), Zhongshan(city), *CLDA* central less derdeveloped areas, such as Liaoning(province), Tangshan(city), Xi’an(city), Wuhan(city), Hunan(province), Hengyang(city), Chongqing(city), Chengdu(city), Yunnan(province), National, southwest China, *WUDA* western or undeveloped areas, such as Gansu, Xinjiang and Shanxi

^b^ *IAQ* interview-administered questionnaire, *SAQ* self-administered questionnaire, *N* not specified questionnaire

^c^ *A* adults, *P* parents, *CS* college students

*NA* not available

**Table 2:** Quality assessment of included studies

Studies	1	2	3	4	5	6	7	Scores	Grade
Huang He, 2013 [33]	1	1	1	1	1	1	1	7	A
Ma Xiaojing, 2013 [12]	1	1	0	1	1	1	1	6	A
He Mei, 2011 [41]	1	0	0	0	1	1	1	4	B
Cui Bo, 2010 [13]	1	1	1	1	1	1	1	7	A
He Xin, 2010 [29]	1	1	1	1	1	1	1	7	A
Xu Jing, 2014 [11]	1	1	1	1	1	1	1	7	A
Feng Suwen, 2010 [46]	1	1	0	1	1	1	1	6	A
Yan Jun, 2013 [43]	1	1	0	1	1	1	1	6	A
Long Xiange, 2011 [21]	1	0	1	0	1	1	1	5	B
Hu Haishan, 2014 [28]	1	1	0	1	1	1	1	6	A
Wu Ying, 2011 [25]	1	0	1	1	1	1	1	6	A
Li Juan, 2011 [14]	1	1	1	1	1	1	1	7	A
Fan Baojian, 2009 [23]	1	0	1	1	1	1	1	6	A
Xiao Wei, 2009 [45]	1	1	0	0	1	1	1	5	B
Wang Xuemin, 2012 [15]	1	1	0	1	1	1	1	6	A
Shao Shujuan, 2013 [17]	1	0	0	1	1	1	1	5	B
Xu Wenyu, 2013 [18]	1	1	1	0	1	1	1	6	A
Ma Dong, 2013 [40]	1	1	1	0	1	1	1	6	A
Zhou Lixia, 2011 [42]	1	1	1	1	1	1	1	7	A
Huang Yanhua, 2014 [39]	1	0	1	1	1	1	1	6	A
Wang Haiqiu, 2011 [31]	1	1	1	0	1	1	1	6	A
Ma Dong, 2012 [30]	1	1	1	0	1	1	1	6	A
Yao Chenglian, 2012 [19]	1	0	0	1	1	1	1	5	B
Xamxinuer Ablimit, 2009 [26]	1	1	0	0	1	1	1	5	B
Xu Lina, 2013 [22]	1	1	0	1	1	1	1	6	A
Zhang Hui,2014 [37]	1	1	1	1	1	1	1	7	A
Yu Jing, 2013 [44]	1	1	0	1	1	1	1	6	A
Guzalnur Abduxkur, 2012 [32]	1	1	0	0	1	1	1	5	B
Cai Jing, 2013 [24]	1	1	0	1	1	1	1	6	A
Li Li, 2010 [27]	1	1	0	1	1	1	1	6	A
Ying Wen, 2014 [35]	1	1	1	1	1	1	1	7	A
Zhang Shaokai, 2013 [38]	1	1	1	1	1	1	1	7	A
Wang Shaoming, 2014 [20]	1	1	1	1	1	1	1	7	A
Yan Hong, 2013 [16]	1	1	0	1	1	1	1	6	A
Li Jing, 2009 [34]	1	1	1	1	1	1	1	7	A
Zhao Fanghui, 2012 [36]	1	1	0	1	1	1	1	6	A
Ayizuoremu · mutailipu, 2015 [64]	1	1	0	1	1	1	1	6	A
Zeng Xiaomin, 2015 [55]	1	0	1	1	1	1	1	6	A
Wang Ling, 2015 [49]	1	1	0	1	1	1	1	6	A
Liu Qiong, 2015 [57]	1	1	1	1	1	1	1	7	A
She Qian, 2015 [59]	1	1	1	1	1	1	1	7	A
Chen Ling, 2015 [50]	1	1	1	1	1	1	1	7	A
Cheng Lihong, 2015 [51]	1	1	1	1	1	1	1	7	A
Zhu Qiaoyang, 2015 [58]	1	0	1	1	1	1	1	6	A
Lei Juhong, 2015 [61]	1	1	1	1	1	1	1	7	A
Zhao Bixia, 2015 [63]	1	0	1	1	1	1	1	6	A
Xie Wenliu, 2015 [48]	1	1	1	1	1	1	1	7	A
Zhou Yanqiu, 2015 [47]	1	1	1	1	1	1	1	7	A
Meng Liping, 2015 [60]	1	1	1	1	1	1	1	7	A
Zou Huachun, 2015 [53]	1	1	1	1	1	1	1	7	A
Zou Huachun, 2015 [52]	1	1	1	1	1	1	1	7	A
Gu Can, 2015 [62]	1	1	0	1	1	1	1	6	A
Wang Wei, 2015 [56]	1	1	1	1	1	1	1	7	A
Abida Abudukadeer, 2015 [65]	1	1	1	1	1	1	1	7	A
Zhang Shaokai, 2015 [54]	1	1	0	1	1	1	1	6	A
Pan Xiongfei, 2015 [66]	1	1	1	1	1	1	1	7	A
Fu Chunjing, 2015 [67]	1	1	1	1	1	1	1	7	A
Hu Shangying, 2105 [68]	1	1	0	1	1	1	1	6	A

For the second index; “data collection strategy”, we considered the study as reasonable when it satisfied one of the following criteria: 1) study purpose and survey contents were explained to participants before the survey, 2) investigators reviewed the questionnaire in terms of the clarity of language and completeness of the questionnaire

For the fourth index, “representativeness of the sample,” we recognized the sample as a good representative when it met one of the following requirements: 1) specific inclusion and exclusion criteria were provided, 2) a reasonable sampling method was used

### Awareness and knowledge of HPV vaccine

**Fig. 2:**
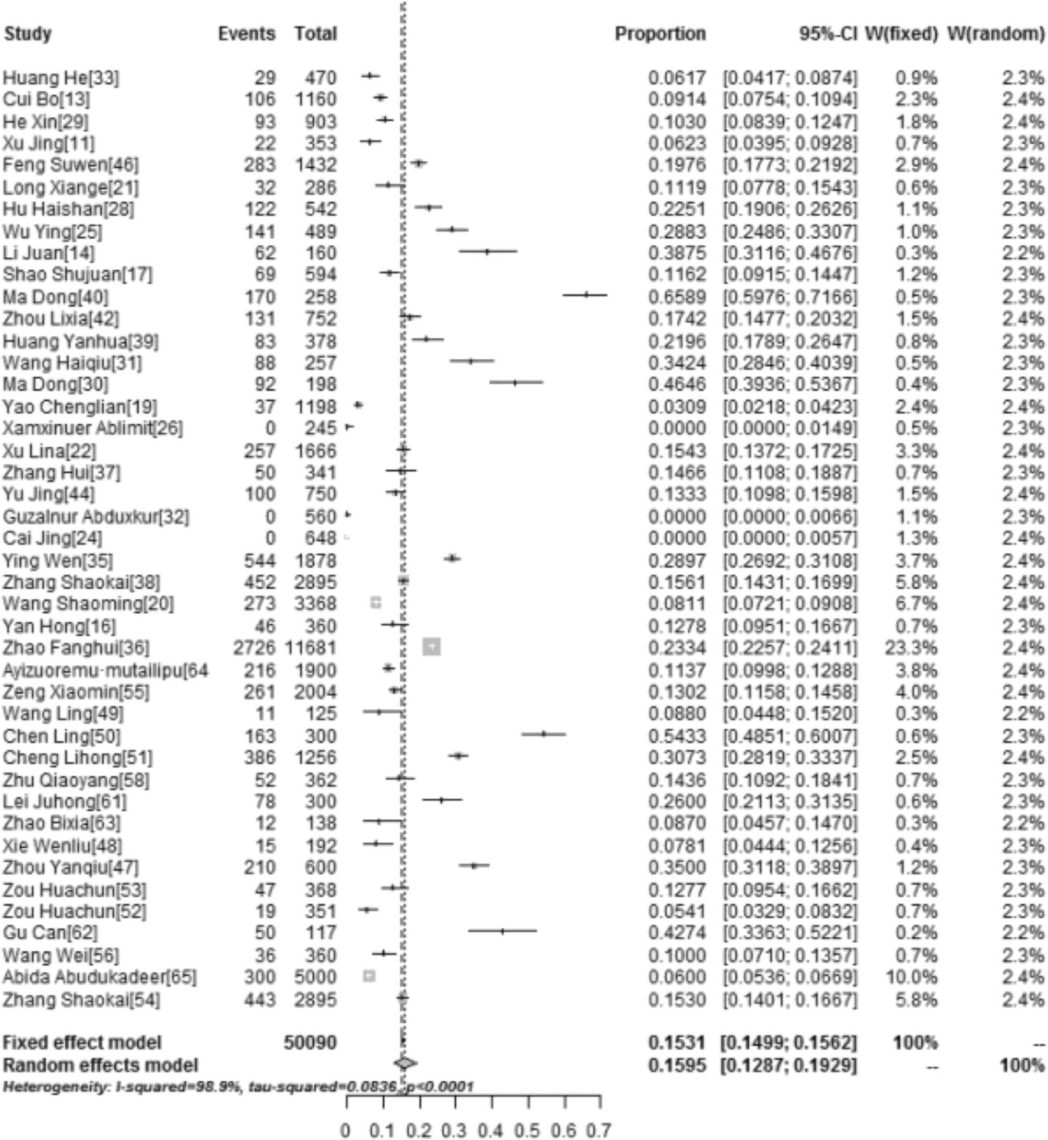
Forest Plot of meta-analysis for HPV vaccine awareness in mainland China, the pooled rate and 95 % CI in the article are from random effects model due to significant heterogeneity which was measured by *I* ^*2*^ statistics

**Fig. 3:**
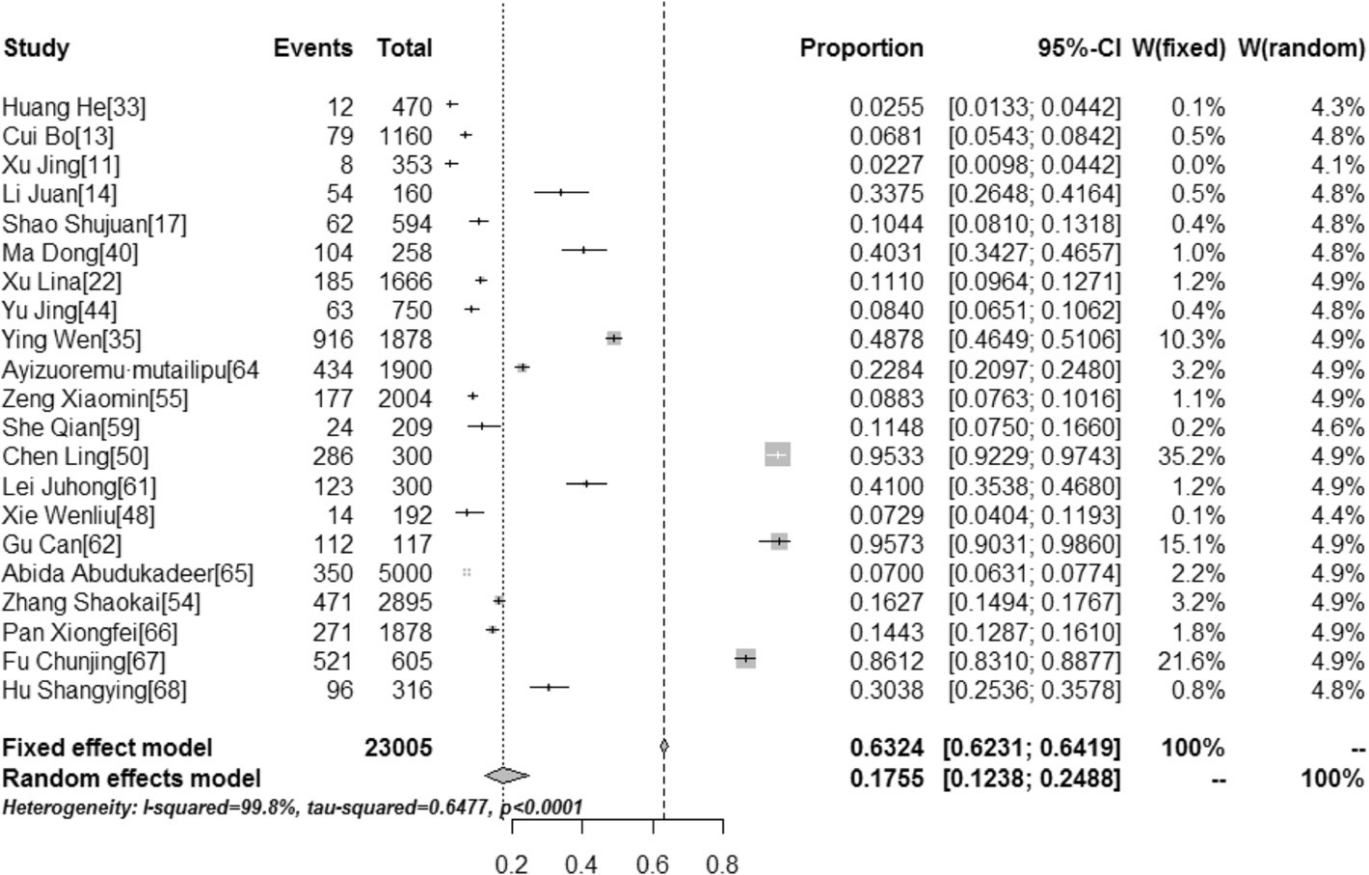
Forest Plot of meta-analysis for HPV vaccine knowledge in mainland China, the pooled rate and 95 % CI in the article are from random effects model due to significant heterogeneity which was measured by *I* ^*2*^ statistics

Awareness and knowledge of HPV vaccination among different populations were reported in 43 and 21 studies, respectively. The pooled awareness rate and knowledge rate concerning HPV vaccination was 15.95 % (95 % CI: 12.87–19.29, *I*^*2*^ = 98.9 %), and 17.55 % (95 % CI: 12.38–24.88, *I*^*2*^ = 99.8 %), respectively (Table 3). Figures 2 and 3 show forest plots of meta-analysis for HPV vaccine awareness and knowledge in mainland China.

**Table 3:** The results of pooled rates of studied items (Supplementary Material: Additional file 1: "Availability of Data and Materials")

Studied items	No. of studies	Pooled rates(95 % CI)	Heterogeneity (*I* ^*2*^,%)	Publication bias(*P* value)
Awareness	43	15.95 (12.87–19.29)	98.9	>0.05
Knowledge	21	17.55 (12.38–24.88)	99.8	>0.05
Acceptability	35	67.25 (58.75–75.21)	99.8	>0.05
Reasons for unwillingness to be HPV vaccinated				
Assumed low risk	14	33.63 (27.50–40.05)	97.2	>0.05
Limited use to date	14	36.31 (29.67–43.22)	97.7	>0.05
Safety	10	50.46 (40.00–60.89)	96.6	>0.05
Efficacy	14	30.18 (23.96–36.79)	97.3	>0.05
Vaccine source	11	32.17 (21.14–43.30)	99.2	>0.05
High price	6	23.72 (13.64–35.59)	98.2	>0.05
Acceptability (for daughters)	12	60.32 (51.25–69.04)	99.2	>0.05
Reasons for unwillingness of parents to vaccinate their daughters				
Limited use to date	4	32.61 (22.03–44.18)	94.5	>0.05
Safety and efficacy	7	68.19 (53.13–81.52)	98.6	>0.05
Vaccine source	7	17.24 (13.87–20.90)	82.8	>0.05
Too young to vaccinate	7	28.37 (13.69–45.90)	99	>0.05

The pooled rate and 95 % CI are from random effects model

### Acceptability of HPV vaccination

**Fig. 4:**
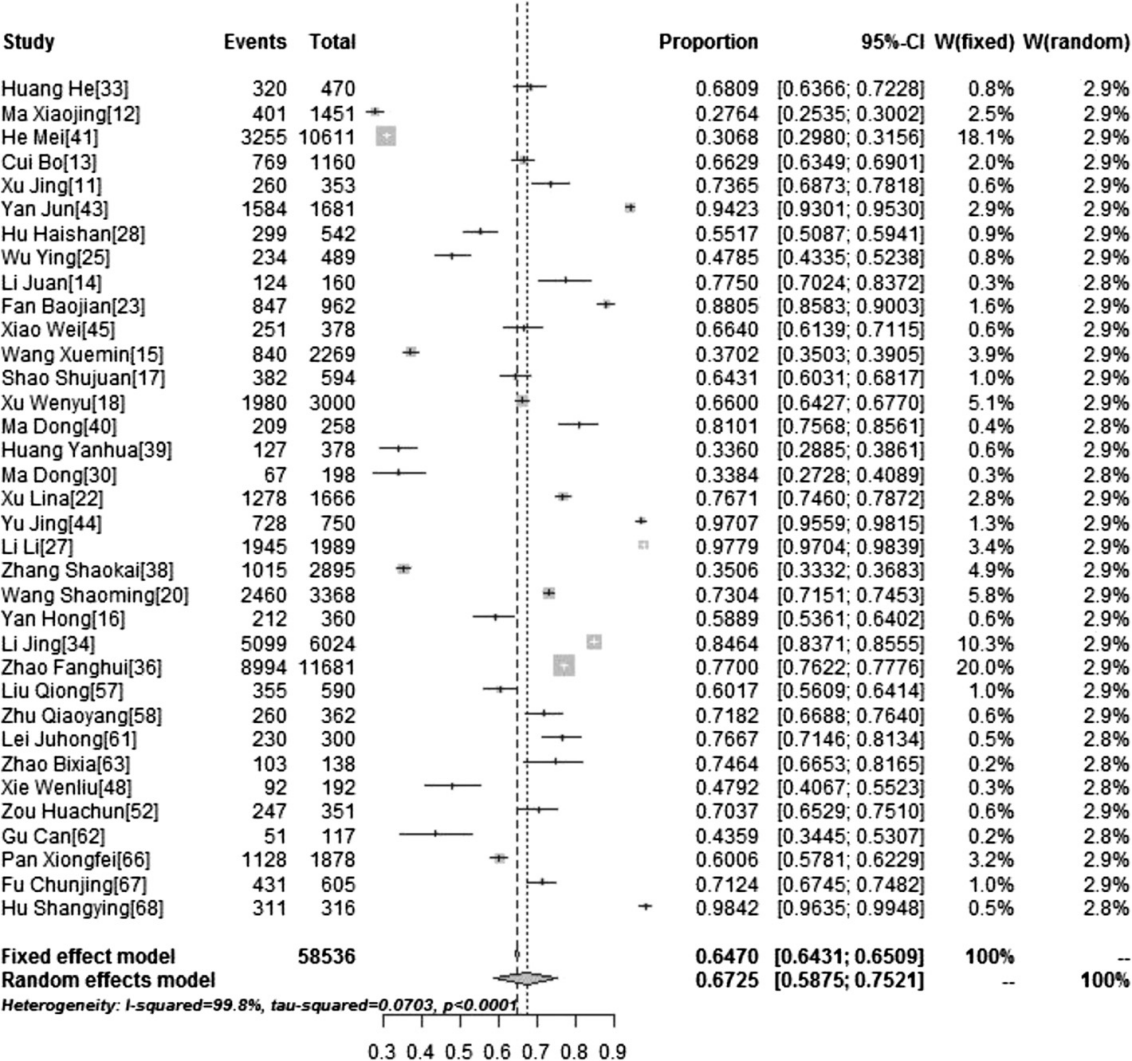
Forest Plot of meta-analysis for acceptability of HPV vaccination(for themselves) in mainland China, the pooled rate and 95 % CI in the article are from random effects model due to significant heterogeneity which was measured by *I* ^*2*^ statistics

**Fig. 5:**
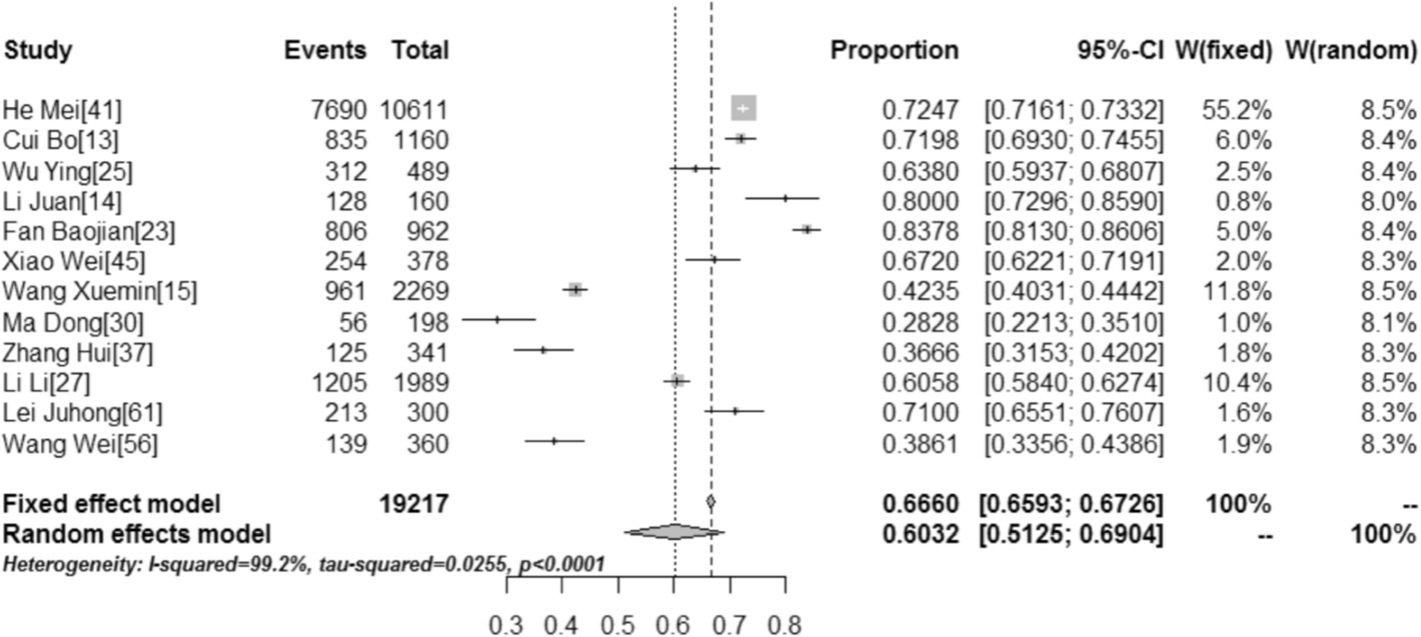
Forest Plot of meta-analysis for acceptability of HPV vaccination(for daughters) in mainland China, the pooled rate and 95 % CI in the article are from random effects model due to significant heterogeneity which was measured by *I* ^*2*^ statistics

We explored the acceptability of HPV vaccination for individuals and their daughters. Thirty-five studies addressed participants’ willingness to be vaccinated, while 12 studies addressed the willingness of parents to get their daughters vaccinated. We found that the willingness of participants to be vaccinated was 67.25 % (95 % CI: 58.75–75.21, *I*^*2*^ = 99.8 %) while their willingness to get their daughters vaccinated was 60.32 % (95 % CI: 51.25–69.04, *I*^*2*^ = 99.2 %) (Table 3). Figures 4 and 5 show forest plots of meta-analysis for acceptability of HPV vaccination (for themselves and their daughters) in mainland China.

### Reasons for unwillingness to be HPV vaccinated

Reasons for the unwillingness of individuals to be HPV vaccinated varied across studies. Nineteen studies explored reasons for participants’ reluctance to HPV vaccination. Among participants who were unwilling to be vaccinated, 33.63 % (95 % CI: 27.50–40.05, *I*^*2*^ = 97.2 %) respondents believed that they had a low risk of developing HPV infection, genital warts, or even cervical cancer. Other respondents were worried about the limited use of HPV vaccine in China (36.31 %; 95 % CI: 29.67–43.22, *I*^*2*^ = 97.7 %). Respondents who were concerned with the safety and the efficacy of HPV vaccination accounted for 50.46 % (95 % CI: 40.00–60.89, *I*^*2*^ = 96.6 %) and 30.18 % (95 % CI: 23.96–36.79, *I*^*2*^ = 97.3 %), respectively. Participants who questioned the source of the vaccine and communicated a concern regarding the high price of the vaccine were 32.17 % (95 % CI: 21.14–43.30, *I*^*2*^ = 99.2 %) and 23.72 % (95 % CI: 13.64–35.59, *I*^*2*^ = 98.2 %), respectively (Table 3).

### Reasons for unwillingness of parents to vaccinate their daughters

Seven studies explored the reasons for participants’ reluctance to get their daughters HPV vaccinated. Among them, 32.61 % (95 % CI: 22.03–44.18, *I*^*2*^ = 94.5 %) respondents were concerned regarding the limited use of HPV vaccine in China to date. Some respondents (68.19 %; 95 % CI: 53.13–81.52, *I*^*2*^ = 98.6 %) doubted the safety and efficacy of the HPV vaccine. Only 17.24 % (95 % CI: 13.87–20.90, *I*^*2*^ = 82.8 %) of the respondents doubted the vaccine source. In addition, 28.37 % (95 % CI: 13.69–45.90, *I*^*2*^ = 99 %) of the respondents considered their children to be too young for vaccination (Table 3).

### Subgroup analysis and meta-regression

A subgroup analysis indicated that the awareness of HPV vaccine differed across sexes (*P* = 0.033), ethnicities (*P* = 0.017), and regions (*P* = 0.031). We observed a higher HPV vaccine awareness among the female population (17.39 %; 95 % CI: 13.06–22.20, *I*^2^ = 98.8 %) and mixed population (18.55 %; 95 % CI:14.14–23.42, *I*^2^ = 98.8 %) relative to the male population (1.82 %; 95 % CI: 0.50–11.20, *I*^2^ = 98.5 %). We also found that populations of mixed ethnicity have lower HPV vaccine awareness (9.61 %; 95 % CI: 5.95–14.03, *I*^2^ = 99.0 %) compared to population of Han (20.17 %; 95 % CI: 16.42–24.20, *I*^2^ = 98.3 %). Among different regions, the HPV vaccine awareness was higher in EDA (17.57 %; 95 % CI: 13.36–22.21, *I*^2^ = 98.0 %) and CLDA (17.78 %; 95 % CI: 12.18–24.19, *I*^2^ = 97.6 %) compared to WUDA (1.80 %; 95 % CI: 0.02–6.33, *I*^2^ = 98.9 %). Subgroup analysis revealed that acceptability to be vaccinated varied among studies conducted using different sampling methods (*P* = 0.022). The acceptability of vaccination among cluster-sampled population (72.45 %; 95 % CI: 52.22–88.76, *I*^2^ = 99.9 %) was higher compared to the convenience-sampled population (53.53 %; 95 % CI: 41.95–64.92, *I*^2^ = 99.5 %). The subgroup analysis showed that acceptability for parents to vaccinate their daughters differed across ages (*P* = 0.014) and sampling methods (*P* = 0.038). General adult population (64.72 %; 95 % CI: 55.57–73.36, *I*^2^ = 99.2 %) was more willing to vaccinate their daughters than parent population (33.78 %; 95 % CI: 26.26–41.74, *I*^2^ = 88.3 %). Randomized sampling method showed a higher acceptability for vaccination of daughters (72.75 %; 95 % CI: 67.66–77.56, *I*^2^ = 92.9 %) compared to cluster sampling method (48.54 %; 32.37–64.88, *I*^2^ = 99.4 %) (Table 4). Meta-regression analysis was also performed but failed to explain the source of heterogeneity.

**Table 4:** The results of subgroup analysis by characteristics of the population

Subgroups	No. of studies	Incidence % (95 % CI)	*I* ^*2*^ (%)	*P* value
Awareness (all studies)				
Age				0.698
CS	12	19.07 (11.72,27.71)	99.0	
A	27	15.14 (11.11,19.68)	99.1	
P	5	15.62 (13.13,18.28)	85.4	
Sex				0.033
F^a^	23	17.39 (13.06,22.20)	98.8	
F, M^a^	17	18.55 (14.14,23.42)	98.8	
M^b^	3	1.82 (0.50,11.20)	98.5	
Sample method				0.504
Randomized	16	18.88 (13.60,24.80)	99.0	
Cluster	16	13.74 (7.66,21.24)	99.1	
Convenience	12	16.03 (12.12,20.36)	97.7	
Ethnicity				0.017
Han	29	20.17 (16.42,24.20)	98.3	
Mixed	15	9.61 (5.95,14.03)	99.0	
Region				0.031
EDA^a^	20	17.57 (13.36,22.21)	98.0	
CLDA^a^	12	17.78 (12.18,24.19)	97.6	
WUDA^b^	5	1.80 (0.02,6.33)	98.9	
Knowledge (all studies)				
Age				0.171
CS	10	40.94 (20.11,63.64)	99.8	
A	11	15.52 (10.22,21.69)	98.7	
P	1	16.27		
Sex				0.841
F	13	27.2 (17.56,38.07)	99.5	
F, M	9	24.51 (10.28,42.45)	99.7	
M	0			
Sample method				0.757
Randomized	11	29.87 (13.02,50.19)	99.7	
Cluster	7	19.16 (12.41,26.98)	98.7	
Convenience	0			
Ethnicity				0.893
Han	14	25.40 (14.41,38.28)	99.4	
Mixed	8	27.46 (13.87,43.62)	99.8	
Region				0.837
EDA	8	19.58 (11.76,28.82)	98.6	
CLDA	9	27.80 (11.97,47.20)	99.7	
WUDA	2	13.96 (2.44,32.70)	99.7	
Acceptability (for themselves)				
Age				0.338
CS	10	71.71 (64.06,78.77)	97.9	
A	24	64.82 (52.80,75.95)	99.9	
P	2	44.92 (26.00,64.63)	98.7	
Sex				0.208
F	21	68.50 (54.40,81.05)	99.9	
F, M	15	61.65 (52.47,70.43)	99.4	
M	0			
Sample method				0.022
Randomized	12	70.42 (63.63,76.79)	98.8	
Cluster^a^	12	72.45 (52.22,88.76)	99.9	
Convenience^b^	12	53.53 (41.95,64.92)	99.5	
Ethnicity				0.939
Han	24	65.14 (53.62,75.83)	99.8	
Mixed	12	66.76 (51.25,80.60)	99.7	
Region				0.407
EDA	14	65.31 (54.68,75.21)	99.2	
CLDA	12	64.10 (46.56,79.87)	99.7	
WUDA	3	82.15 (35.87,99.68)	99.9	
Acceptability (for daughters)				
Age				0.014
CS	1	38.61		
A^a^	10	64.72 (55.57,73.36)	99.2	
P^b^	3	33.78 (26.26,41.74)	88.3	
Sex				0.068
F	8	67.04 (57.46,75.96)	99.2	
F, M	4	46.06 (27.07,65.66)	97.6	
M	0			
Sample method				0.038
Randomized^a^	5	72.75 (67.66,77.56)	92.9	
Cluster^b^	6	48.54 (32.37,64.88)	99.4	
Convenience	3	56.36 (34.63,76.88)	98.8	
Ethnicity				0.253
Han	8	57.84 (47.89,67.48)	99.5	
Mixed	6	61.04 (44.36,76.50)	99.3	
Region				0.689
EDA	6	59.03 (41.51,75.44)	98.9	
CLDA	4	56.36 (37.18,74.60)	99.3	
WUDA	2	51.49 (33.81,68.97)	99.3	

There were significant differences in groups with different letters (*P* < 0.05)

The pooled rate and 95 % CI are from random effects model

*A* adults, *P* parents, *CS* college students

### Publication bias

Egger’s test was performed to assess the publication bias. The results did not show evidence of publication bias (all *P* > 0.05) (Table 3).

## Discussion

This is the first meta-analysis study conducted for the assessment of HPV vaccine related awareness, knowledge and acceptability among the Chinese population. Our meta-analysis identified low awareness (15.95 %) and low knowledge (17.55 %) of HPV vaccine among the Chinese population. The rates were lower compared to many other countries. Studies conducted in Turkey showed that HPV vaccine awareness among undergraduate students in Turkey was 44.5 % [69], while 27.9 % of respondents knew that HPV vaccines can prevent cervical cancer [70]. In addition, the HPV vaccine awareness rates were found to be in the 67.1–71.3 % range in the USA, UK and Australia [71]. The higher awareness rate of HPV vaccine and related knowledge in these countries may be due to the intervention programs and increased media coverage [72–74]. The low level of HPV vaccine awareness may greatly influence its promotion in China. In subgroup analysis, the pooled rate of HPV vaccine awareness was higher among females (17.39 %) and mixed population (18.55 %) compared to the male population (1.82 %). In the Chinese tradition, males play an important role in decision-making in the family, the low awareness of HPV vaccine may influence the acceptability of vaccination for their daughters [53]. We also found that populations of mixed ethnicity have lower HPV vaccine awareness rates (9.61 %) compared to population of Han (20.17 %). In addition, a study in England showed that HPV vaccine awareness was lower among ethnic minority groups (6–18 %) compared to white women (39 %), and that ethnic minorities have lower uptake of vaccination [75, 76]. These findings suggest that potential ethnic inequalities and cultural barriers should be identified for the prevention of cervical cancer [76]. Among different regions, HPV vaccine awareness was higher in EDA (17.57 %) and CLDA (17.78 %) compared to WUDA (1.80 %). In fact, eastern and central areas benefit from abundant healthcare resources and strong economies compared to western or some undeveloped regions. The difference between different geographical areas in China revealed that socio-economic status is a factor that influences the HPV vaccine awareness.

In addition, we found a relative high acceptability of HPV vaccination (67.25 % for themselves and 60.32 % for daughters). However, this rate declines in the high-end of the level across the world (59 % to 100 %) [77–79]. In subgroup analysis, the acceptability to be vaccinated among cluster-sampled population (72.45 %) was higher than convenience-sampled population (53.53 %), and randomized sampling method (72.75 %) showed a higher acceptability for vaccination of daughters compared to cluster sampling method (48.54 %). This is an indication that acceptability of vaccination among the population may be higher if rigorous sampling methods, such as randomized sampling method, are used. Subgroup analysis showed that parental acceptability of vaccination (33.78 %) was lower compared to the general adult population (64.72 %). Moreover, the acceptability rate (33.78 %) was lower compared to similar studies conducted in other countries. A similar study in Sweden reported that 76 % of participated parents were willing to vaccinate their daughters [80]. In addition, studies in Africa showed that parents with good knowledge of HPV vaccine were more willing to vaccinate their children than those with poor knowledge [81]. Population’s attitude and acceptance toward HPV vaccination is an important determinant for the success of HPV vaccine promotion in China in the future, which necessitates, the identification of the main obstacles concerning the acceptability of vaccination among the Chinese population.

The primary obstacles concerning vaccination acceptability for responders were the safety and efficacy of the HPV vaccine. HPV vaccines have been proved safe and efficient against HPV infection [82, 83]. WHO recommended HPV vaccination for both young women and men before the onset of sexual activity [84]. In recent years, many studies have investigated HPV vaccine safety and adverse events. Both of the HPV vaccines are related to high rates of injection site reactions, such as pain, swelling and redness which maybe due to a possible VLP-related (VLP, Virus-like particles) inflammation process [85]. However, these outcomes are usually for a short duration and recovery is quick [86]. Most reported adverse events were mild or moderate in intensity [87–98], and serious vaccination-related adverse events, such as anaphylaxis, are rare [86]. Similarly, other studies reported that there were no vaccine related deaths in the included studies [99]. Furthermore, a review concluded that the prophylactic vaccines against HPV appear safe based on the assessment of reported adverse events by governmental databases and independent researchers [100].

Sufficient scientific evidence has clarified many of the misunderstandings related to vaccine safety, however, the concerns related to vaccination are still increasing [101]. Public confidence in vaccines is particularly important. If vaccination is not trusted, the hesitance to be vaccinated may lead to delay and refusal, resulting in the disintegration of related research and delivery programs, and may even result in disease outbreak [102, 103]. The segmented information from media may amplify vaccine related concerns, resulting in the circulation of anxiety among the public [104]. It is the responsibility of the healthcare providers to rectify the misconceptions related to vaccination among the population, while acknowledging parents' concerns, updating their knowledge on vaccine related health information by paying close attention to the latest scientific research, and allocating sufficient time to instruct the concerned population on vaccine safety [101].

Mainland China has not introduced HPV vaccination into the routine immune vaccination program, experiences from other countries that implemented HPV vaccination program can be taken as an example. In many counties, an organized vaccination program is recommended to increase the vaccination coverage. It is now widely believed that the most urgent public-health issue is to increase HPV vaccination coverage and improve completion of the vaccination schedule, especially among sexually active females [105]. Thus, many studies further explored means to boost vaccination rates. An analysis showed that HPV vaccination rate could not be increased solely by educational intervention. A research conducted in America showed that a provider-centered PICME (Performance Improvement Continuing Medical Education) intervention, which includes repeated communication, focused education, and individualized feedback, proved an effective measure for sustained improvement of vaccination rates [106]. Another study showed obvious differences between adopters and non-adopters via in-depth interviews, emphasizing that vaccinated women benefit from supportive social influences whereas unvaccinated women’s concerns regarding the safety and efficacy of short- and long-term vaccination was influenced by their interpersonal network [107].

Further research to perfect the existing HPV vaccines is needed. Moreover, as a measure of primary prevention, HPV vaccination should be performed alongside cervical screening (secondary prevention) as a clear strategy for the prevention of cervical cancer.

The strength of our analysis is that the evaluation of the recently published papers about HPV vaccination among the Chinese population allowed us to offer evidence-based advice for the implementation of HPV vaccination in Mainland China in future. However, there were some limitations in this study. Obvious heterogeneity existed in the meta-analysis. We tried to perform meta-regression analysis to explain the source of heterogeneity, however, significant heterogeneity remained unexplained after an exploration of the relative factors, such as sampling method and population characteristics. In fact, for observational studies that involve proportions, substantial heterogeneity is a common dilemma [108]. Although a theoretical framework was designed, it is difficult to ensure that all the original studies used rigorous testing and validation for the investigation as previously outlined in real circumstances. These variations and constraints may account, at least partly, towards the observed heterogeneity. In addition, measures of studied factors were inconsistent among studies, and it is difficult to clarify the inconsistencies due to the difference of measurements across included studies or true variability among the population [109].

## Conclusions

In conclusion, this meta-analysis proved low HPV vaccine awareness and knowledge among the Chinese population. HPV vaccine awareness differed across sexes, ethnicities, and regions. However, given the limited quality and number of included studies, future studies with improved design are necessary for the verification of our findings.

## Additional file

Additional file 1: Availability of Data and Materials. (XLS 38 kb)

## Abbreviations

HPV: Human Papillomavirus
PRISMA: Preferred Reporting Items for Systematic Reviews and Meta-Analyses
EDA: Eastern developed areas
CLDA: Central less developed areas
WUDA: Western or undeveloped areas
CI: Confidence interval
I^2^: I-square
VLP: Virus-like particles
PICME: Performance Improvement Continuing Medical Education
